# Comprehensive genome-wide analysis of the GmFRIGIDA gene family in soybean: identification, characterization, and expression dynamics

**DOI:** 10.3389/fpls.2025.1536866

**Published:** 2025-03-10

**Authors:** Song Yu, Yuxuan Wang, Wenwen Ren, Yisheng Fang, Leili Wang, Yifei Zhang, Chengyang Song, Xiao Luo

**Affiliations:** ^1^ College of Agriculture, Heilongjiang Bayi Agricultural University, Daqing, Heilongjiang, China; ^2^ Shandong Key Laboratory of Precision Molecular Crop Design and Breeding, Peking University Institute of Advanced Agricultural Sciences, Shandong Laboratory of Advanced Agricultural Sciences in Weifang, Weifang, Shandong, China

**Keywords:** *Glycine max*, FRIGIDA-LIKE, photoperiod response, gene expression, genome-wide identification

## Abstract

**Background:**

*Frigida* (*FRI*) genes are crucial for regulating flowering time in plants. While the biological importance of the *Frigida-like* (*FRL*) gene family has been recognized in *Arabidopsis*, a systematic analysis of these genes in soybean is lacking. Characterizing *FRL* genes in soybean will help uncover their roles in flowering regulation, offering valuable insights for improving soybean adaptation.

**Results:**

In this study, we identified 16 *Frigida* genes in soybean, naming them based on their relationship to the *FRL* genes in *Arabidopsis thaliana*. These genes are unevenly distributed across thirteen chromosomes. Phylogenetic analysis categorizes Frigida-like proteins from *Arabidopsis*, soybean, and rice into four distinct subfamilies (I–IV). Our findings indicate that eight GmFRLs arose from whole-genome duplication (WGD) events, alongside two tandem duplication events. Gene structure analysis confirmed that all GmFRL members contain Frigida domains. Additionally, promoter analysis revealed numerous cis-acting elements related to photoperiodic response, suggesting their significant role in soybean’s light response mechanisms. RNA-seq data demonstrated variable expression levels of *GmFRL* genes across tissues, including flower, leaf, pod, and seed, and other tissues, while subcellular localization and qPCR analyses further support their vital role in light responsiveness in soybean.

**Conclusion:**

In summary, our comprehensive analysis offers valuable insights into the evolution and potential functions of *GmFRL* genes, emphasizing their significance in photoperiodic responses and establishing a foundation for further research on the GmFRL family.

## 1 Introduction

Seasonal flowering is fundamental to the reproductive success and survival of higher plants. Plants have evolved a complex response of endogenous clues and environmental factors, such as day length and temperature control of flowering time of genetic networks (Choi et al., 2011; Takada et al., 2019; Dean, 2002). *FRI* (*FRIGIDA*) is a key regulator of flowering time and can inhibit flowering without vernalization (Chen et al., 2020). The *FRL* gene encodes a novel protein that lacks structural domains indicative of immediate functions, yet it contains two potential coiled-coil domains (Goff et al., 2002), which are believed to interact with other proteins or nucleic acids. This unique domain activates the expression of *FLC* (*FLOWERING LOCUS C*), which encodes a MADS-box transcription factor that quantitatively suppresses the floral transition by inhibiting flowering pathway integrators, such as *FT* (*Flowering Locus T*) and SOC1 (*SUPPRESSOR OF OVEREXPRESSION OF CONSTANS1*) in *Arabidopsis* (Schmitz and Amasino, 2007). *FRI* acts as a scaffold protein, interacting with FRL1, SUF4, FLX, FES1, UBC1, and CBP20 to form a transcriptional activation complex. This complex recruits chromatin-modifying factors, including the *SWR1* complex and *SET2* homologs, to epigenetically modify the histone methylation levels at the *FLC* locus (Choi et al., 2011; Li et al., 2018).

Recent studies have indicated that *FRI* mediates the activation of the floral repressor *FLC*, thereby negatively regulating flowering time (Zhu et al., 2021). Most rapid-cycling Arabidopsis carry loss-of-function mutations in *FRL*, leading to low levels of *FLC* and rapid flowering in the absence of vernalization (Ding et al., 2013). Studies related to *FRI* genes have been carried out in *A. thaliana* (Risk et al., 2010), *Medicago sativa* (Chao et al., 2013), *Oryza sativa* (Goff et al., 2002), *Populus balsamifera* (Keller et al., 2011) and *Vitis vinifera* (Risk et al., 2010). The *AtFRL* regulates flowering in Arabidopsis, but the orthologs of FRL from *EjFRL* (from loquat) also have the ability to influence *Arabidopsis* flowering. The *FRL* gene is not only related to the regulation of flowering time but also involved in other biological processes related to reproduction, such as embryo development and seed maturation (Hu et al., 2014; Vieira et al., 2019).They found that *PmFRL* may be linked to ABA signal regulators and gibberellin signal regulators, thereby exerting their biological functions during dormancy and flowering in *P. mume* (Li et al., 2021). In tomatoes, they found that Brassinosteroid (BR) regulate tomato flowering through the interaction between SlFRLs and SlBIN2 (Khan et al., 2022). Apple orthologs of *Arabidopsis* genes, FRIGIDA, exhibit similar expression patterns as reported in *Arabidopsis*, suggesting that functional conservation in floral signal integration and meristem determination pathways (Kumar et al., 2016a). These studies reveal the potential important functions of the *FRL* family genes in plant response to regulates flowering.

Soybean serves as a quintessential example of a crop that demonstrates significant sensitivity to photoperiod. The various developmental stages, including the growth, flowering, and maturity periods, as well as the resulting plant morphology, are closely regulated by photoperiodic conditions. The complete genome sequence of the Williams 82 variety (*Glycine max*) was finalized in 2010 and has since been widely used (Schmutz et al., 2010). The primary reference genome assembly, Wm82, which has been in use for the past decade, has undergone significant improvements. Over 3,600 gaps have been closed, adding more than 5 Mbp, and regions previously exhibiting high heterozygosity in the earlier reference assembly have been enhanced. Notably, recent updates to the high-resolution linkage map have significantly strengthened the assembly of the Wm82 reference genome (Valliyodan et al., 2019), incorporating more sequence information and fewer errors. This provides more detailed and accurate gene function annotations, establishing a stronger foundation for the identification and characterization of the *FRL* gene family.

The *FRL* gene family is a key regulatory factor in the control of flowering time in plants. It is essential to identify and analyze *FRLs* on a genomic scale to uncover their molecular functions, which may provide deeper insights into plant development. The identification of *FRLs* has been reported in *Arabidopsis* (Risk et al., 2010) and *Oryza sativa* (Goff et al., 2002), while the phylogenetic and structural characteristics of the *FRL* family have only been studied in *Prunus mume* (Li et al., 2021). Currently, there is no systematic research reporting on the *GmFRL* gene family in soybeans. Therefore, the functional roles of its members within the soybean genome require further investigation. In this study, we identified 16 *GmFRL* genes and conducted a genome-wide analysis of their evolutionary characteristics and biological functions. We examined the phylogenetic relationships, gene structures, conserved motifs, repetitive patterns, cis-element organization, and tissue-specific expression patterns of the *GmFRL* genes. Additionally, since soybean cultivation is not affected by vernalization, the inhibitory role of the *GmFRL* gene in flowering may have potential functions in soybeans. Some evidence suggests that *FRI* does not appear to change photoperiodic responsiveness but rather shifts the response to much later flowering times (Lee and Amasino, 1995). Therefore, we also analyzed the expression response of the *GmFRL* gene to photoperiod in soybeans and its subcellular localization. The systematic analysis of *GmFRL* gene family lays a foundation for further study of its key role in the regulation of soybean light response.

## 2 Materials and methods

### 2.1 Plant materials and growth conditions

The *Glycine max* Williams 82 was used in this study. The seeds were sterilized in 1% sodium hypochlorite for 1 minute, followed by three washes with sterilized water. They were then germinated in a growth chamber for 15 days under LD (16 h light/8 h dark) at 25°C and 60% relative humidity. Fifteen days later, during the seedling stage of soybeans, which is highly sensitive to changes in photoperiod, leaf samples were collected at 0, 4, 8, 12, 16, 20, and 24 hours of Zeitgeber time (ZT). The samples were immediately frozen in liquid nitrogen and stored at -80°C for gene expression analysis (Cao et al., 2015). Three biological replicates were obtained for each time point.

### 2.2 In silico identification of FRIGIDA family genes in *Glycine max*

The Hidden Markov Model (HMM) of FRIGIDA (PF07899) was obtained from PFAM (http://pfam.xfam.org/) and then used as a query to retrieve the soybean proteome sequences. Soybean proteome sequences (Wm82.a4.v1) were downloaded from the phytozome database. To avoid missing FRIGIDA family members, a new HMM based on the resulting sequence is constructed using HMMER software (http://hmmer.org/), and the model is presented as a query sequence (*E values* < 10^-5^), and the sequence data of soybean proteome were retrieved again. After removing the redundant sequences, the SMART online platform (http://smart.embl-heidelberg.de/) checks the remaining sequences to predict the full FRIGIDA domain. The gene encoding a protein with the FRIGIDA domain was identified as a member of the FRIGIDA family. TBtools-II were used to calculate the protein properties of FRIGIDA family members, such as amino acid number, isoelectric point (pI) (Chen et al., 2023). The SOPMA tool (https://www.npsa-prabi.ibcp.fr/cgi-binpsa_automat.pl?page=/NPSApsa_sopma.html) was used to predicted the secondary structure of GmFRL protein, and AlphaFold3 (http://alphafoldserver.com) was used to predicted the tertiary structures of GmFRL proteins.

### 2.3 Evolutionary analysis, chromosomal location, and synteny analysis

ClustalW is used for multiple sequence alignment of all GmFRL proteins, and a phylogenetic tree of GmFRL proteins was constructed using MEGA 11.0 (Hall, 2013; Thompson et al., 2003; Tamura et al., 2021), and a comprehensive phylogenetic tree that includes *Arabidopsis*, soybean, and rice. Both phylogenetic trees were constructed using the maximum likelihood (ML) algorithm with 1000 Bootstrap repeats (Kumar et al., 2016b), Gene duplication events and synteny analysis (*Glycine max* vs. *Arabidopsis*; *Glycine max* vs. *Glycine max*) were performed using the default parameters of Tbtools-II software (Chen et al., 2023).

### 2.4 Structural characterization, conserved motif analysis, and cis-acting elements

The exon/intron structure of each *GmFRL* gene was analyzed by TBtools-II (Chen et al., 2023). The conserved motifs of all GmFRL proteins were analyzed by MEME tool (http://meme-suite.org/tools/meme). Conserved domains within all *GmFRL* proteins were identified using the CD-Search tool (https://www.ncbi.nlm.nih.gov/cdd/) from the NCBI database. Cis-acting elements in the promoter sequences (upstream of 2000 bp) of the *GmFRL* gene family were predicted using the PlantCare website (http://bioinformatics.psb.ugent.be/webtools/plantcare/html/). Tbtools-II is used to visualize results (Chen et al., 2023).

### 2.5 Subcellular localization of GmFRL proteins

CELLO v.2.5 (http://cello.life.nctu.edu.tw/) was used to predict the subcellular localization of all *GmFRL* proteins. Subsequently, four *GmFRLs* were cloned and transiently overexpressed in tobacco leaves for subcellular localization experiments to validate the prediction results. The *GmFRL* genes were amplified and subsequently ligated into the fusion expression vector pSuper1300-MAS-EGFP following digestion, leading to the successful construction of the expression vector. The recombinant plasmids were then transformed into Agrobacterium strain GV3101. Both the Agrobacterium containing the pSuper1300-MAS-GmFRL01/04/09/13-EGFP expression vector and those harboring the empty control vector pSuper1300-MAS-EGFP were cultured, and the bacterial cells were harvested by centrifugation at 4000 rpm for 15 minutes, followed by the removal of the supernatant. Subsequently, 1 mL of tobacco transformation solution (OD600 = 0.7–1.0) was added to resuspend the Agrobacterium. After resuspension, the tobacco leaves were injected following a 2-hour incubation at room temperature or 28°C. Approximately 2–3 days post-injection, the lower epidermis of the tobacco leaves was peeled off, and the subcellular localization of the fused protein was observed using confocal microscopy, with images captured simultaneously.

### 2.6 Analysis of expression patterns of *GmFRL* genes

According to the RNA-seq data (TPM) of *GmFRL* extracted from SoyMD (Yang et al., 2024), the expression pattern of *GmFRL* genes in different tissues of soybean was studied. Transcriptomic data on soybean were obtained from the National Center for Biotechnology Information (NCBI) publicly accessible database (Accession number: GSE94228). After removing adapter sequences and low-quality reads from the RNA-seq data using fastp (v.0.23.0) (Chen et al., 2018), we aligned the cleaned RNA-seq data to the Wm82.a4.v1 genome using HISAT2 (v.2.1.2) (Kim et al., 2015) with default parameters. We then quantified and normalized the data using StringTie (v.1.3.5) (Pertea et al., 2015) with default settings. The heat map function in Tbtools-II was used for further expression analysis (Chen et al., 2023).

### 2.7 RNA extraction and qPCR analysis

Total RNA was extracted from 15-day-old seedlings with TRIzol Reagent (Invitrogen) and reverse transcribed by MMLV reversetranscriptase (Promega). Quantitative Real-time PCR (q-PCR) was performed with a SYBR Green PCR Master Mix kit. Analysis was performed using the Applied Biosystems StepOnePlus real-time PCR system. Whole plant seedlings from wild-type on the same place were collected separately at the same time. Three independent experiments were conducted. Relative transcript levels were normalized to *GmACT11*. The reaction and the calculation of relative expression levels were performed as described previously (Miura et al., 2007). The qRT-PCR was carried out as described previously (Iqbal et al., 2020; Tong et al., 2009).

## 3 Results

### 3.1 Identification of *GmFRL* genes in soybean

A total of 16 *GmFRL* genes were identified from the soybean genome (Wm82.a4.v1) within the Phytozome v13 database. Based on their homology with members of *AtFRL* family, the 16 *GmFRL* family genes were designated as *GmFRL1* to *GmFRL16* reflecting their homology with members of the *AtFRL* family (**Table 1**). The physicochemical properties of *GmFRL* genes were predicted as shown in the **Table 1**, including the number of amino acids, molecular weight, and theoretical isoelectric point (pI). The full lengths of the GmFRL proteins varied between 519 amino acids (GmFRL3) and 1297 amino acids (GmFRL16), with molecular weights ranging from 56.80 kDa to 152.26 kDa. Additionally, the isoelectric points varied from 5.90 (GmFRL5) to 9.13 (GmFRL10). Overall, the pI of GmFRL family proteins showed significant differences. All proteins were hydrophilic, as reflected by Grand Average of Hydropathicity less than 0.

**Table 1:** Analysis of the physicochemical properties and primary and secondary structure of 16 *GmFRL* genes.

Gene name	Gene ID	Protein length (aa)	Molecular Weight (kDa)	pI	Instability Index	Aliphatic Index	Grand Average of Hydropathicity	Hh	Extended Strand (Ee)	Random coil (Cc)	Predicted location
*GmFRL01*	*Glyma.08G325700*	546	60.42	6.56	52.24	81.45	-0.46	0.37	0.11	0.52	nucl
*GmFRL02*	*Glyma.18G081400*	549	60.51	7.06	55.46	77.14	-0.48	0.41	0.08	0.52	nucl
*GmFRL03*	*Glyma.02G297300*	519	56.80	8.91	56.26	79.94	-0.39	0.47	0.08	0.45	nucl
*GmFRL04*	*Glyma.03G096300*	544	60.99	6.42	48.17	85.20	-0.47	0.49	0.15	0.35	nucl
*GmFRL05*	*Glyma.03G096400*	546	61.30	5.90	52.30	81.52	-0.52	0.53	0.12	0.36	nucl
*GmFRL06*	*Glyma.16G079100*	546	61.09	5.95	45.15	81.15	-0.51	0.50	0.13	0.37	nucl, cyto
*GmFRL07*	*Glyma.05G101800*	553	61.71	5.92	51.03	82.26	-0.61	0.50	0.09	0.40	nucl
*GmFRL08*	*Glyma.17G165100*	553	61.62	6.96	51.50	82.98	-0.58	0.54	0.09	0.37	nucl, cyto
*GmFRL09*	*Glyma.05G231700*	520	57.64	8.92	53.78	73.44	-0.54	0.49	0.07	0.44	nucl
*GmFRL10*	*Glyma.08G039100*	520	57.61	9.13	51.56	72.69	-0.55	0.49	0.08	0.43	nucl
*GmFRL11*	*Glyma.10G248000*	530	58.38	6.74	52.40	86.00	-0.31	0.44	0.09	0.46	nucl
*GmFRL12*	*Glyma.20G146100*	524	57.63	6.38	50.02	84.58	-0.33	0.43	0.10	0.47	nucl
*GmFRL13*	*Glyma.04G203400*	601	65.76	6.05	37.02	85.41	-0.36	0.45	0.12	0.43	nucl
*GmFRL14*	*Glyma.06G162100*	592	64.88	7.56	40.57	76.33	-0.46	0.45	0.11	0.44	nucl
*GmFRL15*	*Glyma.11G109500*	960	110.75	8.47	51.22	82.54	-0.74	0.62	0.07	0.31	nucl
*GmFRL16*	*Glyma.12G015800*	1297	152.26	7.87	45.17	76.92	-0.93	0.70	0.06	0.24	nucl

**Figure 1:**
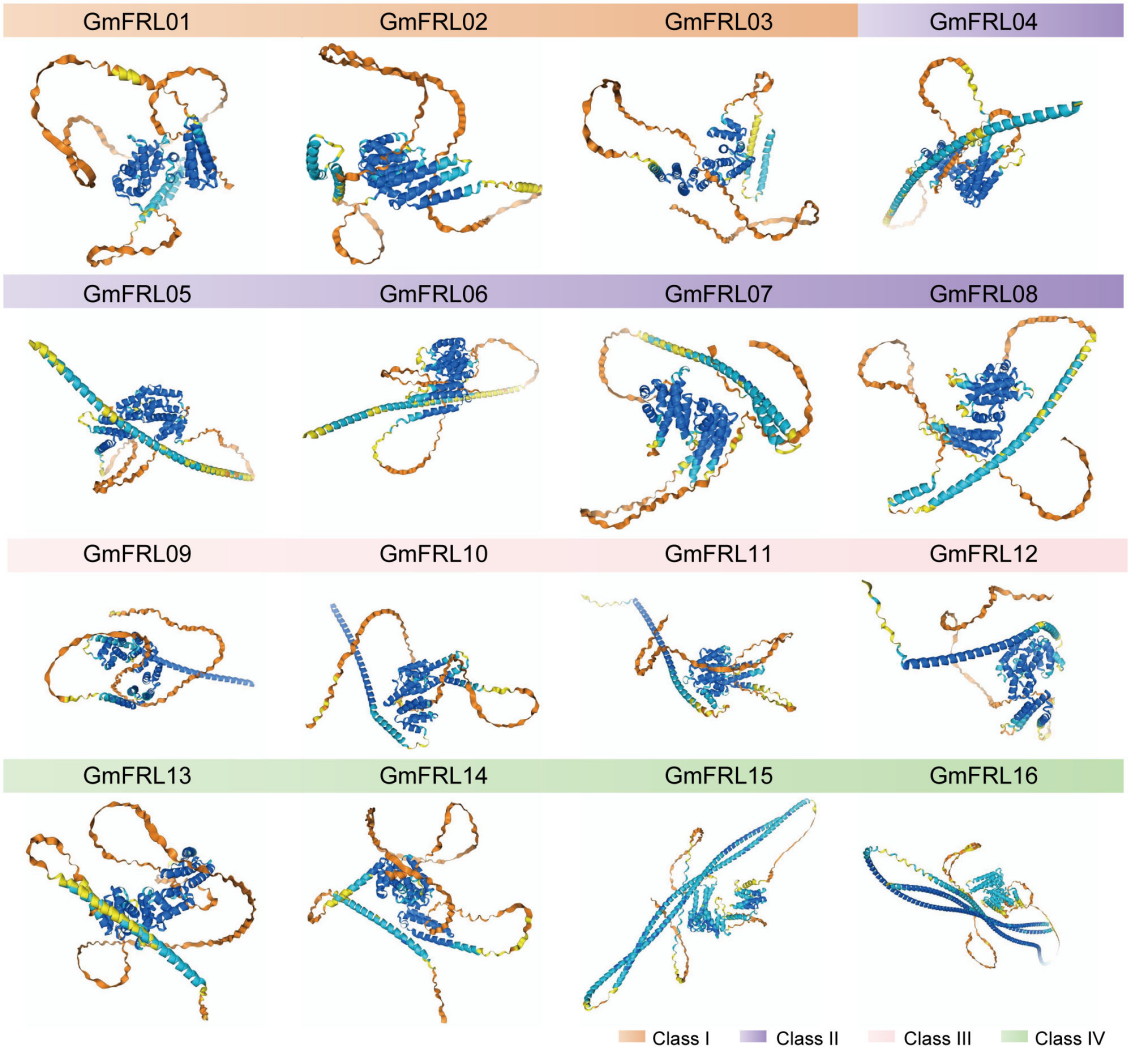
Tertiary structure model of GmFRL proteins. The different colored labels the different class of GmFRL proteins.

According to secondary structure prediction by the SOPMA tool, the GmFRL proteins were found to encompass α-helix, extended strand, and random coil secondary structural elements (**Table 1**). The α-helix content was the highest overall, constituting 37.36%-70.01%, followed by random coil, constituting 23.52%-52.01%, while the extended strand was the lowest, constituting 6.48%-12.82%. Prediction using the Alpha Fold3 tool revealed that the tertiary structures of same subfamily of GmFRL proteins were largely similar (**Figure 1**), which was consistent with the gene structure analysis outcomes.

### 3.2 Chromosomal distribution and expansion patterns of *GmFRL* genes

**Figure 2:**
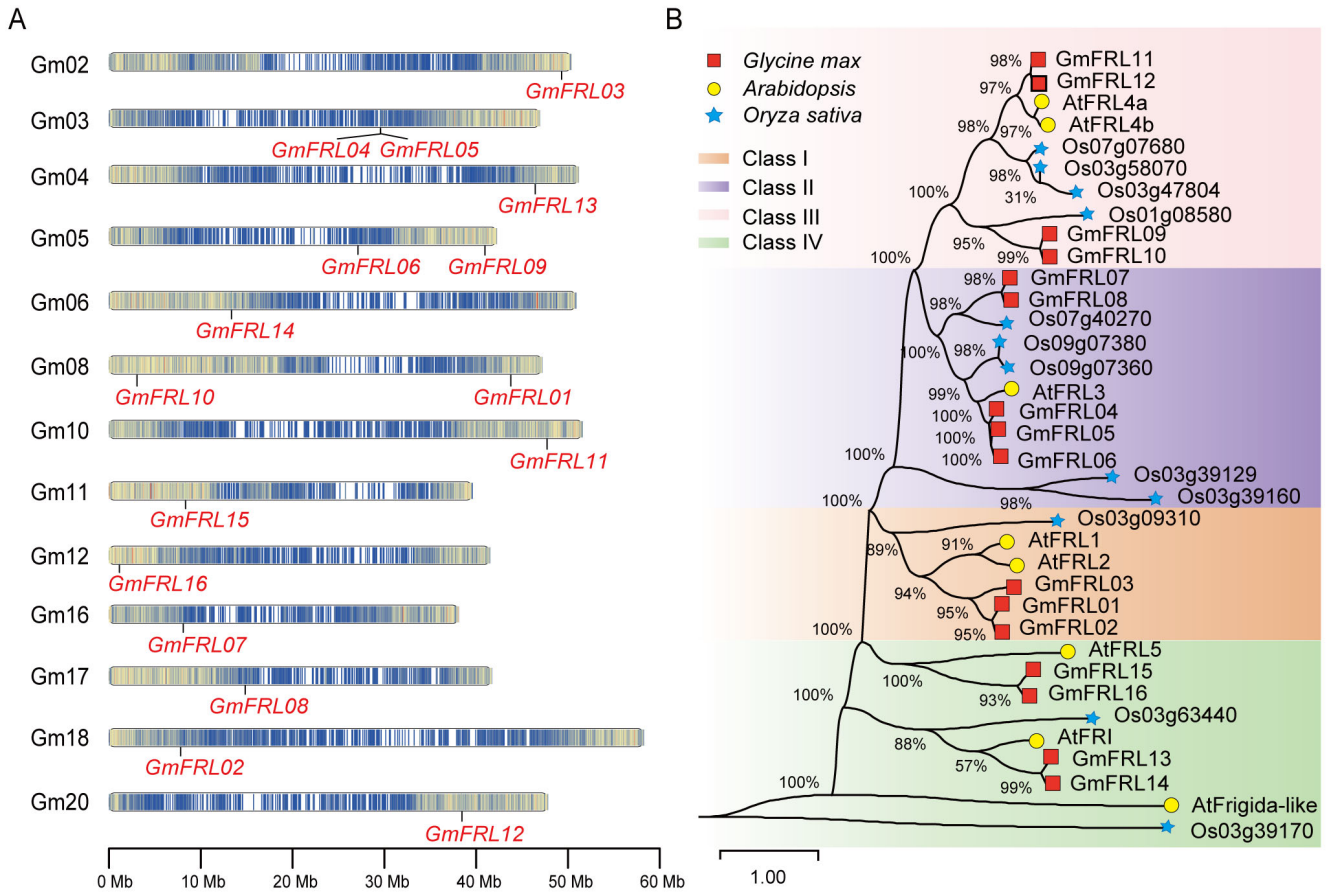
Genomic distribution and phylogenetic analysis of the *GmFRL* genes. **(A)** Distribution of *GmFRL* genes across chromosomes. The scale at the bottom indicates the length of the chromosomes, with the chromosome numbers displayed on the left side of each chromosome. **(B)** Phylogenetic analysis of Frigida-like proteins from soybean, *Arabidopsis* and rice. Phylogenetic trees were constructed using the maximum likelihood (ML) algorithm with 1000 bootstrap repetitions.

**Figure 3:**
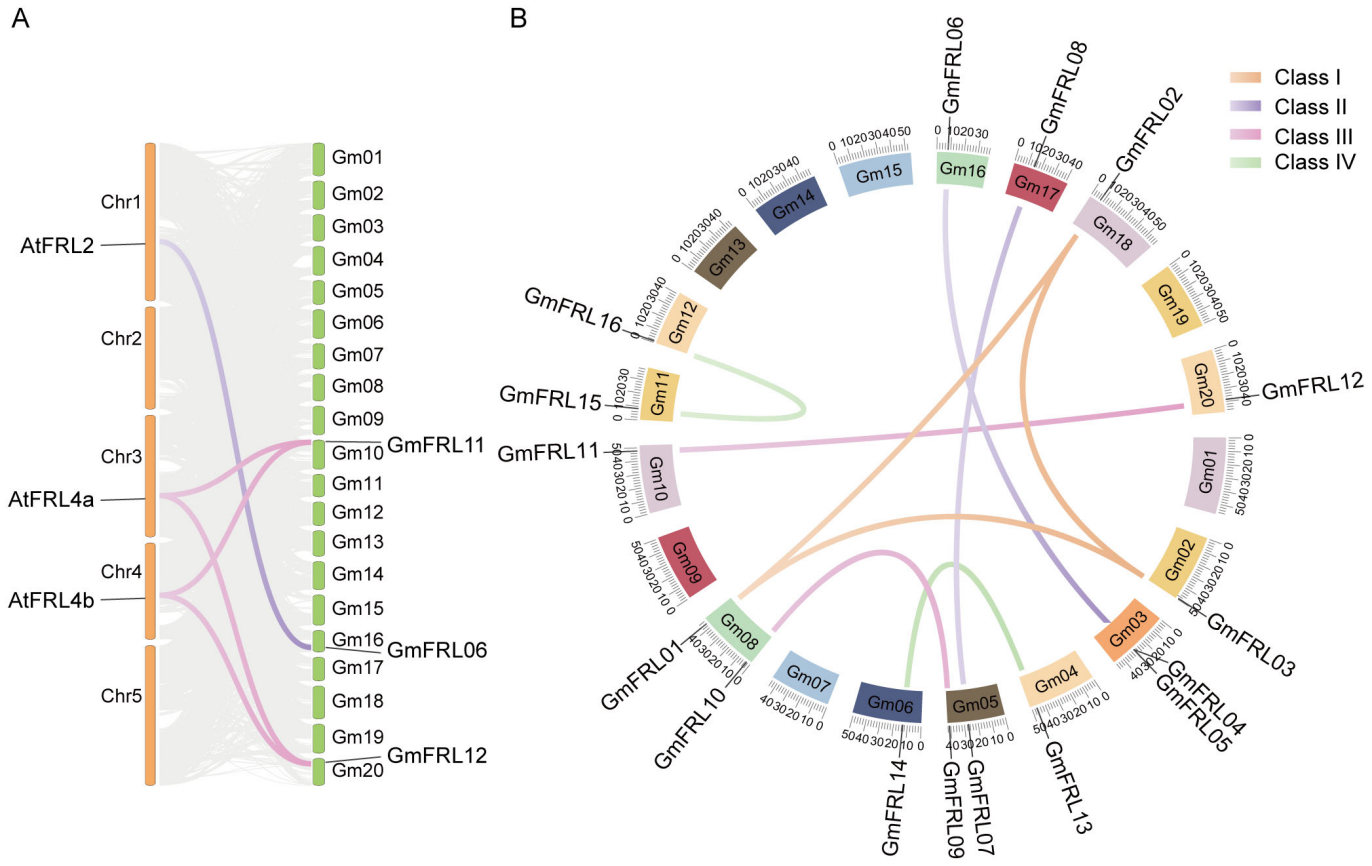
Collinear analysis of GmFRL proteins and FRL proteins from different plants. **(A)** The chromosomal distribution and Syntenic relationships prediction of *Glycine Max* and *Arabidopsis thaliana FRL* genes. **(B)** Distribution and synteny analysis of *GmFRL* genes. The soybean chromosomes are resented in different-colored partial circles. The different colored labels the different class of *GmFRLs*.

TBtools software was used to illustrate the physical locations of *GmFRL* genes on soybean chromosomes. The results indicated that the 16 *GmFRL* genes were unevenly distributed across 13 chromosomes (**Figure 2A**). Specifically, chromosomes 3, 5, and 8 each contained two *GmFRL* genes each, while the remaining chromosomes carried only one gene. Notably, chromosomes 1, 7, 9, 13, 14, 15, and 19 did not harbor any *GmFRL* genes. These findings suggest that the distribution of *GmFRL* genes across soybean chromosomes is not uniform. Co-linearity analysis of the *GmFRL* genes revealed two pairs of tandemly repeated sequences among the 16 identified *GmFRL* genes. To investigate the evolutionary relationship between *AtFRL* and *GmFRL*, a syntenic map of the genomes of *Arabidopsis* and *Glycine max* was visualized using a circos plot (**Figure 3A**). The syntenic map exhibited a linear relationship between *GmFRL11*, *GmFRL12*, *AtFRL4a* and *AtFRL4b*. Furthermore, *GmFRL06* and *AtFRL2* displayed a linear relationship, suggesting that they share homology and may serve similar functions.

The duplication events among *GmFRL* genes were analyzed, focusing on segmental and tandem duplications, to gain insights into their expansion within the soybean genome (**Figure 3B**). The results indicated that the gene pair *GmFRL04* and *GmFRL05* expanded through tandem duplication. In contrast, the gene pairs *GmFRL01*/*02*/*03*, *GmFRL07*/*08*, *GmFRL04*/*06*, *GmFRL09*/*10*, *GmFRL11*/*12*, *GmFRL13*/*14*, and *GmFRL15*/*16* underwent expansion through segmental duplication. These findings suggest that segmental duplication is the primary driving force behind the significant expansion of *GmFRL* genes in the soybean genome. According to the KaKs_Calculator 3.0, the Ka/Ks values of the tandemly repeated *GmFRL* sequences (*GmFRL04*/*GmFRL05*) were 0.1679 (Ka = 0.0584, Ks = 0.3479), which is less than 1. This suggests that purifying selection has acted on these genes during the process of evolution. In other words, *GmFRL* genes are highly conserved and evolve slowly.

### 3.3 Phylogenetic analysis of the GmFRL proteins

Phylogenetic analysis of 36 FRL proteins from 3 species of different affinities was performed that including 16 from *Glycine max*, 8 from *Arabidopsis thaliana*, and 12 from *Oryza sativa*. The results of the phylogenetic analysis indicated that these FRL proteins were classified into four distinct subgroups (I~IV) (**Figure 2B**). Each subgroup contains 3 to 5 members. The presence of both soybean FRL proteins and AtFRL proteins within the same subgroup suggests a possible conservation of function among dicot species. Generally, members within the same subgroup may have similar functions, which aids in our understanding of the potential biological functions of GmFRLs. In subgroup I, three members of the GmFRL family (GmFRL1/2/3) clustered with two members of the AtFRL family, including AtFRL1 and AtFRL2. AtFRL1 and AtFRL2 play crucial roles in regulating *FLC* expression in *Arabidopsis*, with their functions dependent on the genes that affect the chromatin structure of the FLC locus (Emami and Kempken, 2019). In subgroup II, five members of the GmFRL family clustered with an AtFRL family protein named AtFRL3, which can regulate the photoperiod pathway in *Arabidopsis* (Li et al., 2019). In a previous study, GmFRL07 was identified as potentially participating in the regulation of soybean growth stages, based on the strong correlation peak SNP and LD blocks of four significant SNPs (Gm5_27111367, Gm11_10629613, Gm11_10950924, Gm19_34768458) (Li et al., 2019).

### 3.4 Gene structures and protein motifs of GmFRL family

**Figure 4:**
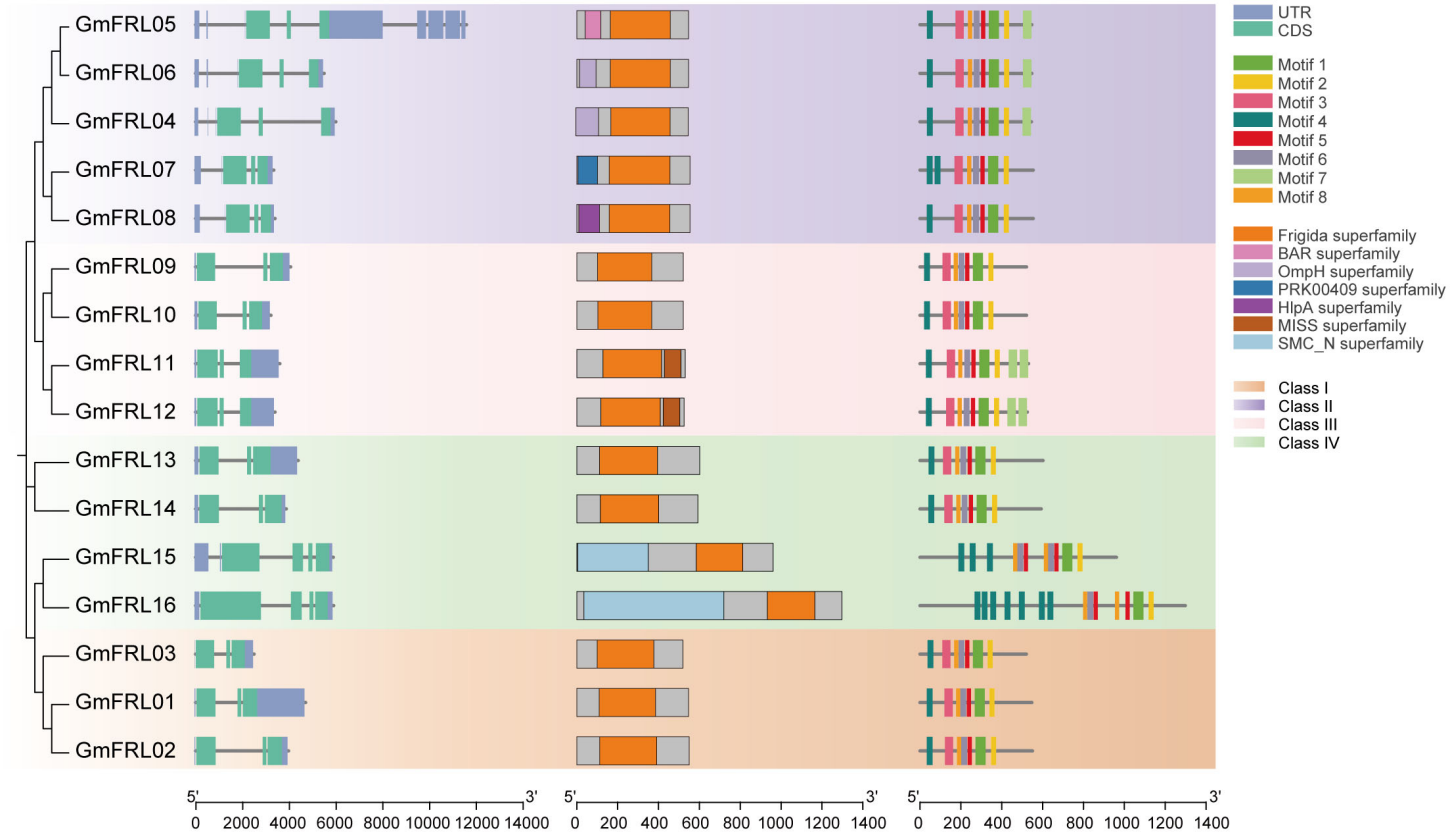
Analysis of the intron and exon compositions of *GmFRL* genes, as well as the conserved domains and motifs of GmFRL proteins, is conducted in the context of their phylogenetic relationships.

Through the screening of sequences and annotation files, we delineated the gene structures for the *GmFRL* family (**Figure 4**). The number of introns within the *GmFRL* genes spanned from 2 to 8, whereas the number of exons fluctuated between 3 and 4. Among the *GmFRL* genes, *GmFRL03* demonstrated the maximum number of introns, totaling eight. Furthermore, eight genes possessed two introns (*GmFRL01, GmFRL04, GmFRL07, GmFRL06, GmFRL08, GmFRL09, GmFRL10, GmFRL15*, and *GmFRL16*), whereas the remaining *GmFRL* genes possessed either three or four introns.

Conserved domain analysis revealed that all members of four subfamily possess Frigida domains, implying a potential conservation of function. To further elucidate the structural diversity of the GmFRL proteins, we identified a total of eight conserved motifs utilizing the MEME suite (**Figure 3**, **Supplementary Figure S1**). As illustrated in **Figure 3**, all GmFRL proteins exhibited motif-1. Although the types and numbers of motifs among members of the same subfamily were comparable, some variations in motif patterns were observed among specific members. In summary, the members of the same subfamily displayed similar structures and conserved motifs, thereby reinforcing the reliability of the constructed phylogenetic tree.

### 3.5 Cis−acting elements in *GmFRL* promoters

**Figure 5:**
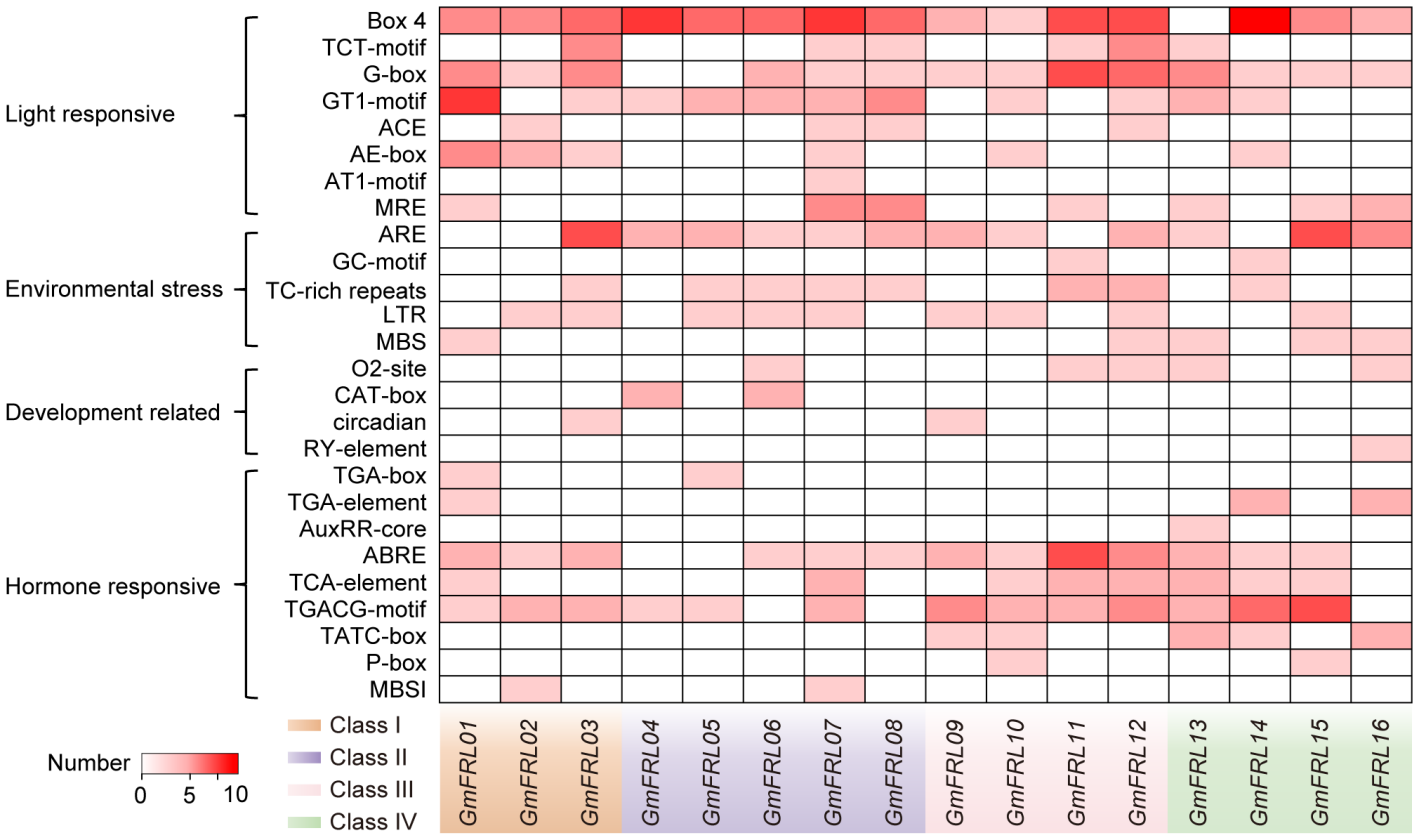
Predicted cis-elements in the promoters of *GmFRL* genes. Types and numbers of cis-elements in the promoter regions of *GmFRL* genes.

Cis-acting elements in the promoter region play a crucial role in how plants respond to growth factors and environmental stresses by regulating gene expression through transcription (Walther et al., 2007). In this study, in addition to the abundant core promoter (TATA box) and enhancer elements (CAAT box), four types of cis-regulatory elements were identified: light-responsive, stress-responsive, hormone-responsive, and development-responsive elements (see **Figure 5**). Among these, light-responsive elements were the most abundant. The number of stress-related response elements and hormone-responsive elements was roughly equivalent, while development-related elements were the least prevalent. Nearly all *GmFRL* genes contained various types of light-responsive elements. For example, *GmFRL01* included the GT1-motif (a light-responsive element), the AE-box (part of a module for light response), and Box 4 (a conserved DNA module associated with light responsiveness). These findings suggest that *GmFRL* genes may play a significant role in light responsiveness in soybeans. Further analysis of hormone-related response elements in the promoters of *GmFRL* family members indicates that these elements can be classified into categories associated with auxin, gibberellic acid (GA), abscisic acid (ABA), salicylic acid (SA), and methyl jasmonate (MeJA). This categorization underscores their potential significance in plant hormone signaling and stress responses.

### 3.6 Expression patterns in different soybean tissues and subcellular localization of *GmFRL* family

**Figure 6:**
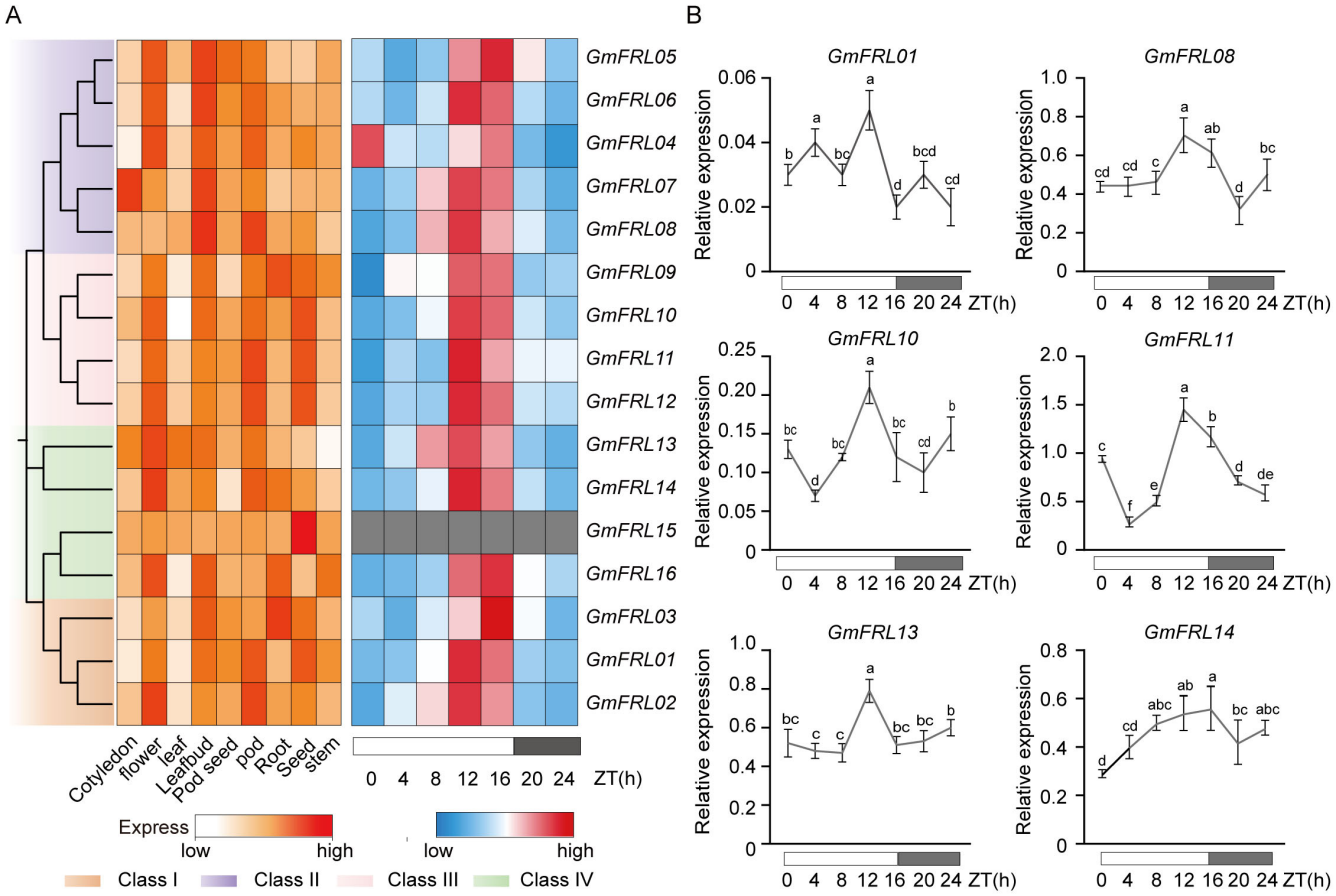
Expression pattern analysis of *GmFRL* genes. **(A)** Expression levels of *GmFRL* genes at different tissues. And the expression levels of *GmFRL* genes in LD. **(B)** Relative expression levels of *GmFRL* genes in response to LD light. The soybean cultivar Wm82 was cultured for 15 days, and the relative expression levels of *GmFRL* genes were measured at 0h, 4h, 8h, 12h, 16h, 20h, and 24h under LD. The *GmACT11* gene served as the internal control. The data represented the mean ± SD of three independent biological repetitions.

To understand the potential functions of *GmFRL* family, The RNA-seq data (TPM) to study *GmFRL* gene expression patterns in different soybean tissues, including shoot apical meristem (SAM), root, root hair, stem, leaf, flower, pod, nodule, and seed (**Figure 6A**). The heatmap illustrates the expression of 16 *GmFRL* genes across 9 tissues. The results indicate that all 16 *GmFRL* genes are expressed in at least one tissue, with most family members showing high expression levels in flowers and seeds. There are notable differences in tissue-specific expression among the different subfamilies of *GmFRLs*, Class II *GmFRLs* exhibit specific expression in flowers and leaf buds, while Class III *GmFRLs* are specifically expressed in pods and seeds. Additionally, compared to other *GmFRL* family members, certain *GmFRL* genes, such as *GmFRL03*, and *GmFRL09* are specifically expressed in leaf buds; *GmFRL06*, *GmFRL08*, and *GmFRL16* are specifically expressed in leaf buds; *GmFRL13* exhibits significantly higher expression levels in flowers, leaves, and leaf buds; and *GmFRL15*, are virtually non-expressed in all tissues except for seeds. This observation may indicate that some *GmFRLs* play a potential role in regulating flowering or seed development in plants.

**Figure 7:**
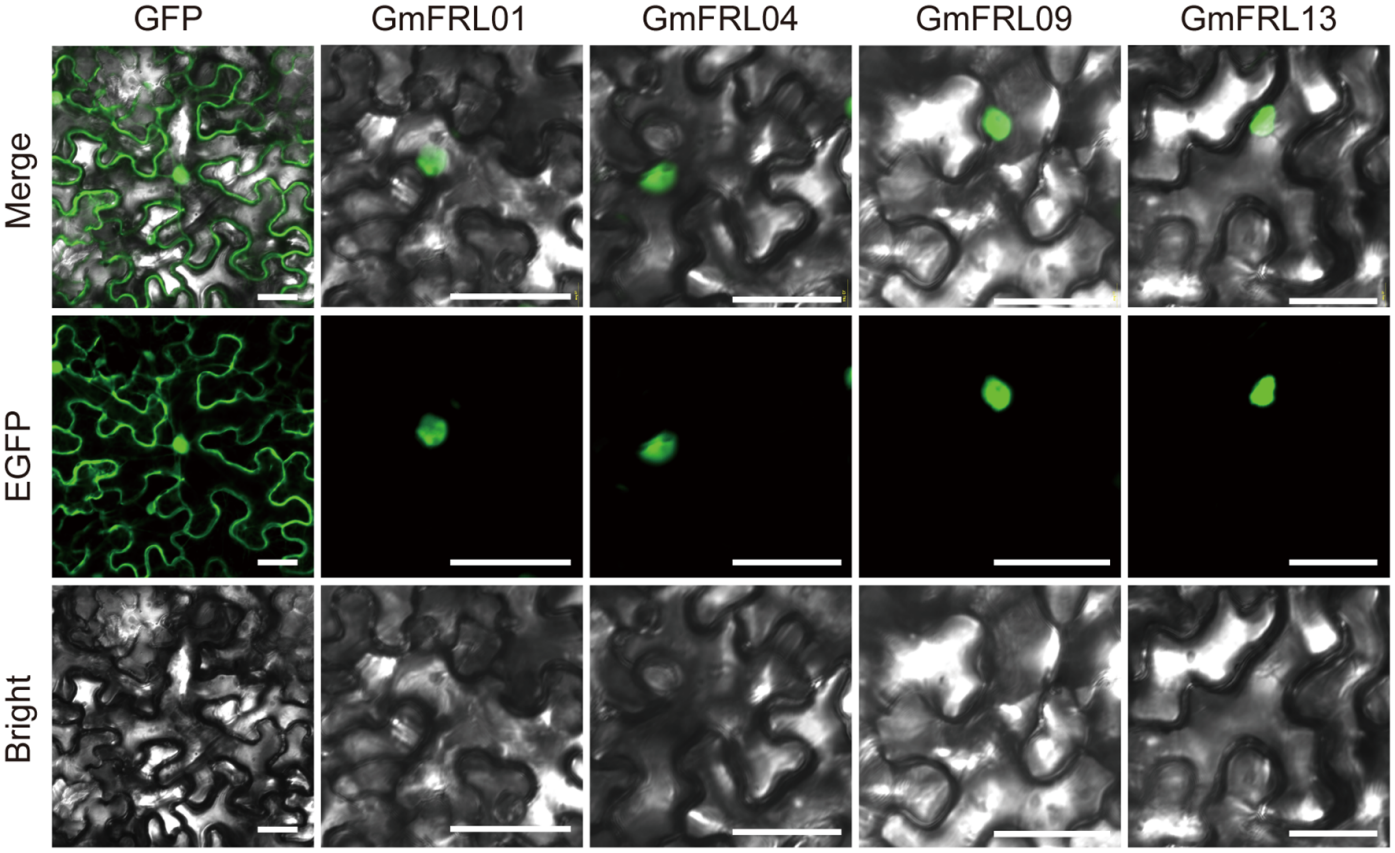
Subcellular localization analysis of *GmFRL* genes. eGFP indicates the fusion protein; Bright, bright field; Merge, merged GFP and Bright. Bars 50μm.

In addition, we utilized CELLO to predict the subcellular localization of all members, with the results indicating that they are primarily localized in the nucleus (**Table 1**). To further validate the subcellular localization of GmFRL proteins, we conducted transient expression experiments with GFP-GmFRL01/04/09/13 constructs in *Nicotiana benthamiana*. The results indicated that the green fluorescence of GmFRL01/04/09/13 was distinctly visible in the nucleus (**Figure 7**). This observation leads us to hypothesize that GmFRL proteins may be primarily localized in the nucleus.

### 3.7 Expression patterns of *GmFRL* genes in different light condition

Previous studies have emphasized the significant role of phytochrome-mediated regulation in flowering time. RNA-seq transcriptome data from soybean leaves at multiple time points (0, 4, 8, 12, 16, 20, and 24 hours) under long-day (LD) light was analyzed. The expression of *GmFRLs* exhibits a rhythmic pattern across multiple cycles (**Supplementary Figure S2A**). The results from a single cycle demonstrate that, except for *GmFRL15*, which is virtually non-expressed in leaves, all other Class I-IV *GmFRLs* exhibit a rhythmic expression pattern, with higher expression levels observed around ZT16, approximately 12 hours after light exposure or at dusk (**Figure 6A**; **Supplementary Figure S2B**). To further investigate whether the expression of *GmFRL* genes is indeed influenced by circadian rhythms, RT-qPCR was performed under LD conditions to analyze the relative expression levels of *GmFRL* genes across different time points. The results indicated that several *GmFRL* genes (*GmFRL01*, *GmFRL08*, *GmFRL10*, *GmFRL11*, *GmFRL13*, and *GmFRL14*) exhibited higher expression levels at ZT12 or around dusk at ZT16 (**Figure 6B**). Based on these findings, we propose that certain *GmFRL* members may play a potential role in the response to light under long-day conditions in soybean.

## 4 Discussion

*FRL* genes play an important role in the process of plant growth and development. To date*, FRL* family have not been identified or analyzed in *Glycine max*. The continuously updated whole-genome sequencing of soybean provides a valuable resource for bioinformatics analyses of various gene families within this species (Wang et al., 2023). In this study, we identified a total of 16 *GmFRLs* from *Glycine max*, a significantly greater number than the 8 *AtFRLs* identified in *Arabidopsis* (Michaels et al., 2004). Furthermore, we conducted comparative analyses of FRL-like proteins from different plant genomes, including mosses, ferns, and various angiosperms (**Supplementary Figure 3**). The evolutionary analysis of the *FRL* family indicates that the FRL structure was established prior to the divergence of terrestrial plants and algae, as we identified proteins with FRL configurations in the unicellular alga *Chlamydomonas reinhardtii*. Comparative analyses of *FRLs* among mosses, rice, and Arabidopsis reveal that the *FRL* family underwent independent expansions during the early evolution of terrestrial plant lineages, following the divergence of angiosperms and bryophytes, and throughout the diversification of each angiosperm lineage. The most pronounced expansions were observed in the lineages of soybean and maize, where the number of *FRL* members is nearly double or even four times that found in *Arabidopsis*.

Gene duplication is a major driving force for the expansion of gene families and the evolution of novel functions, such as adaptation to stress and induction of disease (Cannon et al., 2004; Panchy et al., 2016; Vision et al., 2000). The presence of two or more genes on the same chromosome indicate a tandem duplication event, while two or more genes present on different chromosomes reveal a segmental duplication event. Tandem duplication and segmental duplications have been considered as the main duplication patterns for gene family expansion (Kong et al., 2007; Yu et al., 2005). Previous studies indicate that the soybean genome experienced two rounds of segmental duplication in its evolutionary history, occurring approximately 13 and 59 million years ago (Mya). This has led to a highly duplicated genome, in which nearly 75% of the genes are present in multiple copies across various genomes (Schmutz et al., 2010). The genes that arise from these genomic duplication events provide the raw material for the generation of new genes, which in turn promotes the development of new functions, thereby facilitating the expansion of gene families and functional evolution. A comprehensive review of existing literature on genomic repeat events in soybean and its ancestral species, *Glycine soja*, indicates that 8 (50%) of the *GmFRL* genes are derived from whole genome duplication (WGD) events in wild soybean (Du et al., 2012), while 4 (25%) of the *GmFRL* genes are classified as singletons. These findings suggest that the *GmFRL* gene family in soybean has undergone significant gene duplication throughout its evolutionary history and has been retained through multiple WGD events. Furthermore, this study identifies that 2 of the 16 *GmFRL* genes have experienced tandem duplication, which may be associated with an early legume duplication event that occurred approximately 28 million years ago. This observation underscores the contribution of tandem repeats to the expansion of the *GmFRL* gene family. In summary, segmental duplication emerges as the primary mechanism driving the expansion of the *GmFRL* family, occurring in conjunction with tandem duplication events among certain members, thereby facilitating the overall proliferation of the *GmFRL* gene family. The Ka/Ks ratio is a measure used to examine the mechanisms of gene duplication evolution after divergence from their ancestors (Salih et al., 2016). The Ka/Ks ratio provides insight into the selection pressure acting on amino acid substitutions: a Ka/Ks ratio < 1 indicates purifying selection, a ratio of 1 suggests neutral selection, and a ratio > 1 indicates positive selection. The Ka/Ks values for the tandem duplicate *GmFRL* genes are less than 1, suggesting that these genes have undergone negative selection. This result reflects a slow evolutionary rate and significant conservation within this gene family.

In addition, the molecular weights of different GmFRL proteins exhibit variability, indicating potential differences in their structure and composition, which suggests that their functions may also differ. A phylogenetic tree analysis showed that members of the *GmFRL* family were classified into 4 distinct subgroups. Some GmFRL proteins and AtFRL proteins belong to the same subfamily, perhaps this part of the GmFRL proteins have a genetic structures and conserved motifs, which may contribute to their crucial biological functions. In this study, we identified the number of introns for 16 *GmFRL* members; *GmFRL03* had 8 introns, others genes had 2-4 introns (**Figure 3**). Introns were considered to be a necessary way to acquire new gene functions and preferred to rise at the earlier stages of gene expansion and gradually diminish over time (Roy and Gilbert, 2006; Rose, 2018; Roy and Penny, 2007; Iwamoto et al., 1998).

Expression pattern analyses can provide valuable insights into the potential functions of genes. RNA-seq data indicate that all 16 *GmFRL* genes were expressed in at least one tissue, with the majority of gene family members exhibiting high expression levels in flowers and seeds, suggesting their essential roles in these developmental stages. The tissue-specific expression of *GmFRL* genes varies among different classes, which may imply functional diversification. Some *GmFRLs* may have specific functions in particular tissues and could be involved in the soybean life cycle. Environmental signals are typically sensed by leaves, while flowers develop from primordia that form on the sides of the *SAM* (Searle et al., 2006). In *Arabidopsis*, the localized expression of *FRI* in the phloem and leaves activates *FLC*, thereby delaying flowering (Searle et al., 2006). At the same time, the spatial expression of *FRI* in roots may generate a mobile signal that is transmitted from the roots to the shoot apex, antagonizing *FT* signaling to further delay flowering (Kong et al., 2019). Therefore, the specific tissue expression patterns of *GmFRLs* may be related to the mechanism by which *FLC* regulates flowering in a spatially dependent manner. *FRI* itself encodes a large protein that cannot move over long distances, but by upregulating *FLC* expression, *FRI* can function in specific tissues, including the phloem, leaves, shoot apical meristem, and roots, to delay flowering (Kong et al., 2019).

Previous studies have identified that a major determinant of flowering time in natural variants of *Arabidopsis thaliana* is the *AtFRI* gene. *AtFRI* functions by upregulating the expression of the floral repressor *FLC*, thereby establishing a vernalization requirement and promoting a winter annual growth habit (Zhu et al., 2021). In other research, they analyzed the localization of *AtFRI in vivo* of the *Arabidopsis.* Like many other co-transcriptional regulators (Sabari et al., 2020; Bienz, 2020) they found that FRI–GFP forms nuclear condensates, which were increased in size and number after cold exposure (Zhu et al., 2021). Other research showed that *FRI* function is also suppressed by mutations in the *FRI* homologs *FRL1* and *FRL2*, and these FRI-related proteins, therefore, may all form a complex *in vivo*. these *FRI* interactors both influence *FLC* capping but fall into different groups of *FRI* suppressors: those that are specific for *FRI* and those that suppress *FLC* up-regulation more generally. *FRI* suppressors with apparently different specificities might appear to influence a common mechanism through intimate connection of the co-transcriptional processes linking 5^’^ capping, 3^’^ end formation, nuclear export, and transcriptional elongation (Geraldo et al., 2009). In our study, we analyzed the subcellular localization of the *GmFRL* gene family. The subcellular localization results revealed that four genes (*GmFRL01*, *GmFRL04*, *GmFRL09*, and *GmFRL13*) exhibited a surprising and intriguing observation: the GmFRL01/04/09/13-GFP fusion proteins localize primarily in nuclear condensates. This finding suggests that certain *GmFRL* genes may share functional similarities with *AtFRI* in *Arabidopsis*, possibly acting as transcriptional activators. However, functional analysis of the *GmFRL* family is still in its early stages, and these results warrant further investigation through gene cloning and expression analysis in future studies.

In *Arabidopsis*, the winter annual growth habit is conferred by *FRIGIDA* (*FRI*) and *FLC*. FRI encodes a plant-specific scaffold protein and functions dominantly to upregulate *FLC* expression to a high level that inhibits flowering (Choi et al., 2011). RNA-seq data and qPCR results show that most *GmFRL* genes have an expression pattern that is upregulated during midday, and downregulated in dusk. These results reveal that *GmFRL* genes might play potential roles in the photoperiod pathway, in addition to responding to cold signals to regulate flowering time in soybean. However, within the molecular network regulating flowering in plants, the expression of the *FLC* gene is directly or indirectly regulated by various key factors through different pathways. This study focused solely on exploring and validating the photoperiodic response of *GmFRLs* under long-day conditions. Further research is needed on the light-responsive expression patterns of *GmFRL*, as well as its effects on plant flowering under combined regulatory conditions with light response.

## 5 Conclusions

This study presents a comprehensive and systematic analysis of the *GmFRL* gene family utilizing bioinformatics approaches. A total of 16 members of the soybean *FRL* gene family were identified, and the amplification and functional differentiation of the *GmFRL* gene family were explored. The expression profiles generated under varying photoperiods, along with the results from subcellular localization studies, underscore the potential roles of *GmFRL* genes in the photoperiodic responses of soybean. The extensive bioinformatics and expression analyses conducted on the *GmFRL* genes significantly enhance our understanding of their functions in light responses and the regulation of flowering time. Collectively, these findings establish a robust theoretical framework and provide a valuable reference for future investigations into the associated functions and regulatory mechanisms of these genes.

## Data Availability

Publicly available datasets were analyzed in this study. This data can be found here: https://www.ncbi.nlm.nih.gov/geo/query/acc.cgi?acc=GSE94228.

## Supplementary material

Supplementary Figure 1 Motif logo of GmFRL conserved domains.

Supplementary Figure 2 The expression levels of *GmFRL* genes. **(A).** According to the RNA-seq data, the expression levels of *GmFRL* genes in LD within 48h. **(B).** The relative expression levels of the remaining *GmFRL* genes were measured at 0, 4, 8, 12, 16, 20, and 24 hours under long-day (LD) conditions.

Supplementary Figure 3 Inventory of *FRL* genes in different plant genomes.
