# Supplementary figures and images for: Comprehensive genome-wide analysis of the GmFRIGIDA gene family in soybean: identification, characterization, and expression dynamics
Source: Front Plant Sci. 2025 Mar 10;16:1536866. doi: 10.3389/fpls.2025.1536866 (PMC11932152; doi:10.3389/fpls.2025.1536866)

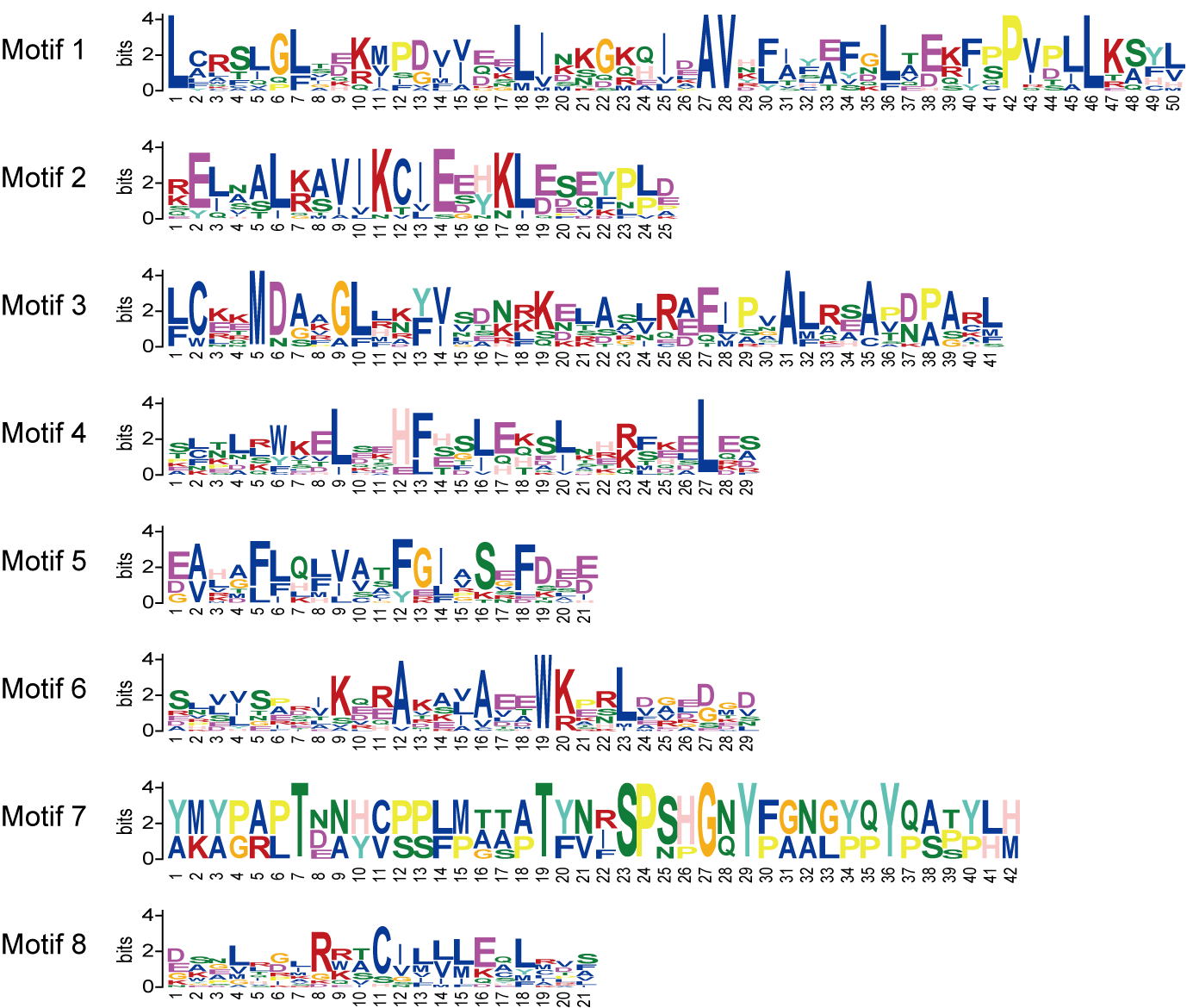

Supplement: Supplementary Figure 1 — Motif logo of GmFRL conserved domains. [file Image1.tif]

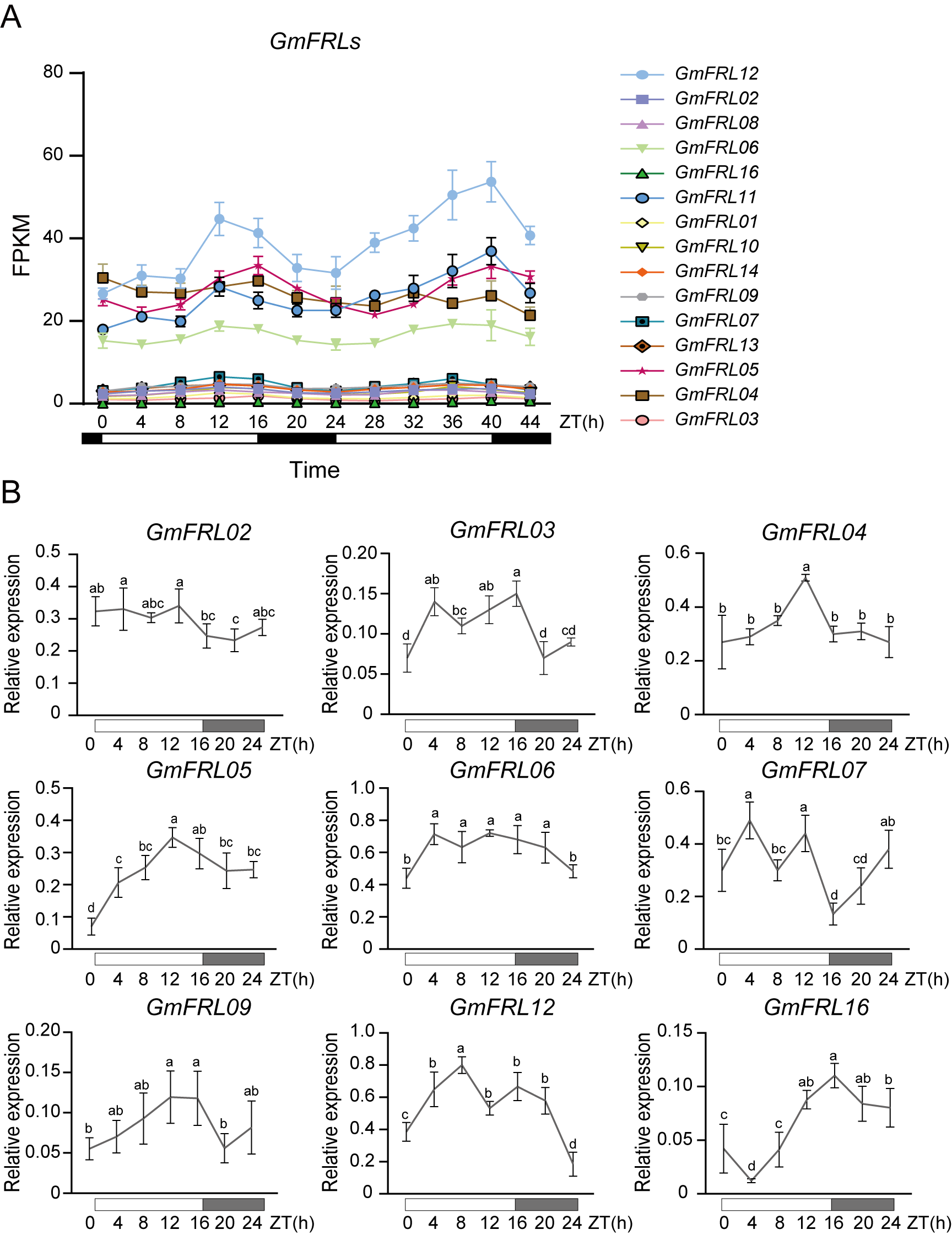

Supplement: Supplementary Figure 2 — The expression levels of GmFRL genes. (A). According to the RNA-seq data, the expression levels of GmFRL genes in LD within 48h. (B). The relative expression levels of the remaining GmFRL genes were measured at 0, 4, 8, 12, 16, 20, and 24 hours under long-day (LD) conditions. [file Image2.tif]

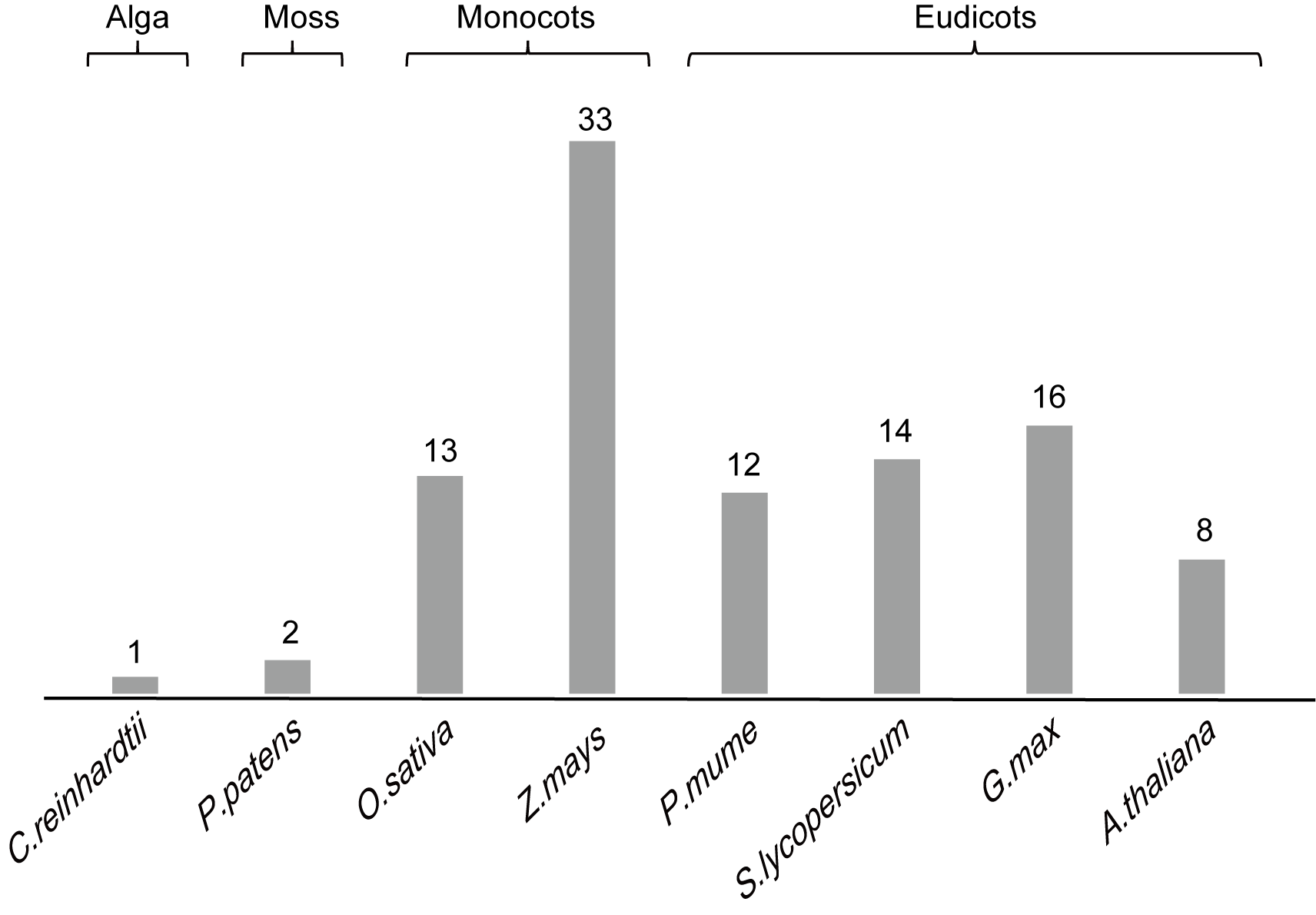

Supplement: Supplementary Figure 3 — Inventory of FRL genes in different plant genomes. [file Image3.tif]
